# Virtual On-Call: Use of Low-Fidelity Simulation to Improve Preparedness for Practice

**DOI:** 10.7759/cureus.73916

**Published:** 2024-11-18

**Authors:** Molly M Nichols, Amy Radcliffe, Allen Daniel

**Affiliations:** 1 Medical Education and Simulation, Maidstone and Tunbridge Wells National Health Service (NHS) Trust, Kent, GBR

**Keywords:** bleep, junior doctor confidence, low fidelity, on-call handover, on-call shift, preparedness training, residency preparedness, simulation in medical education, surgical-education, undergraduate medical student

## Abstract

Background

Many newly qualified doctors feel unprepared for clinical practice. The literature identifies themes including difficulties with clinical reasoning, emergency management, handover, and prioritization of tasks. Although there is an expected level of anxiety for newly qualified doctors, this appears to be amplified with respect to the first on-call shifts that encompass these themes.

Materials and methods

Virtual on-call (VOC) is a low-fidelity, ward-based simulation for senior undergraduate medical students designed to simulate an on-call in a supported environment with high psychological fidelity. Sessions were provided across two hospital sites for students to attend voluntarily. Three simulation sessions were created, each composed of five medical and surgical scenarios of differing complexity. Students responded to simulated bleeps (pager messages) and attended relevant wards to find patient notes and complete paper-based tasks. A student-led handover concluded the simulation followed by facilitator-led structured feedback and debrief. Students completed pre- and post-session questionnaires collecting quantitative and qualitative feedback. Facilitators received feedback on their teaching. A total of 30 resident doctors volunteered to teach, and 39 students attended at least one session.

Results

Pre-session questionnaires highlighted that 91% of respondents (n=32) felt scared/nervous/petrified about the idea of their first on-call. Prior to the first VOC session, the baseline assessment highlighted a lack of confidence among medical students regarding on-call working. Post-session results (session one) showed statistically significant increases in confidence in all the themes assessed (paired t-test with statistical significance considered at p<0.05). Forty-seven percent of first-session participants (n=14) felt positive about on-call working after attending VOC. Students who completed multiple sessions continued to have significant increases in their overall confidence levels between sessions. All students who attended three sessions were left feeling positive about their first on-call (n=2).

About 95% (n=38) reported a constructive learning environment which was useful to improve preparedness for practice and time management skills. Although students reported finding the experience stressful at times, they remarked how it was beneficial to have "the opportunity to practice a wide range of skills while in an on-call simulation, how to manage acute situations, how to prioritize, and how to escalate to a senior." They reported feeling "more confident holding the bleep, finding guidance, and seeking guidance."

Conclusion

This program fills an unmet educational need. Feedback was overwhelmingly positive, displaying significantly increased confidence in multiple skills associated with being a safe and successful on-call doctor. We hope that the confidence gained from the on-call program will translate to improved practice when the participants qualify as doctors with a positive impact on patient care.

## Introduction

Preparedness for practice has been defined as "an interplay of dynamic and complex constructs from competence, self-confidence, capability, and adaptability" [1]. It has been noted that beyond achieving competencies, individuals must be able to integrate and apply such skills in complex and changing environments [1].

It is widely acknowledged that many newly qualified doctors feel unprepared for clinical practice [2,3] with specifically heightened anxiety and lack of confidence regarding the first on-call shifts. Such shifts encompass several non-clinical and clinical factors with associated increased responsibility and uncertainty [3]. The literature displays recurring themes highlighting a range of on-call tasks that new doctors find particularly challenging: handover, responding to emergency bleeps, task prioritization, management of acute conditions, and clinical reasoning/diagnostics [4,5].

This work was previously presented as a meeting abstract at the International Association for Health Professions Education (AMEE) Meeting in August 2023.

## Materials and methods

Teaching program design

To address this ubiquitous educational need, a group of resident doctors (previously termed junior doctors) of varying grades designed a low-resource, face-to-face simulation program for medical students - 'virtual on-call' (VOC). The sessions were designed to assess student competencies of the skills listed above, while emulating the complex and changing environment associated with on-call working, with the aim to improve their confidence and preparedness for practice.

Three sessions were developed, each comprising five medical/surgical case-based scenarios with associated tasks of differing complexity. Example scenarios include: warfarin prescribing, death verification, review of patients with gastrointestinal bleeding/ bowel obstruction/ sepsis, and post-fall review (see Figure 8 in Appendices).

**Figure 1:**
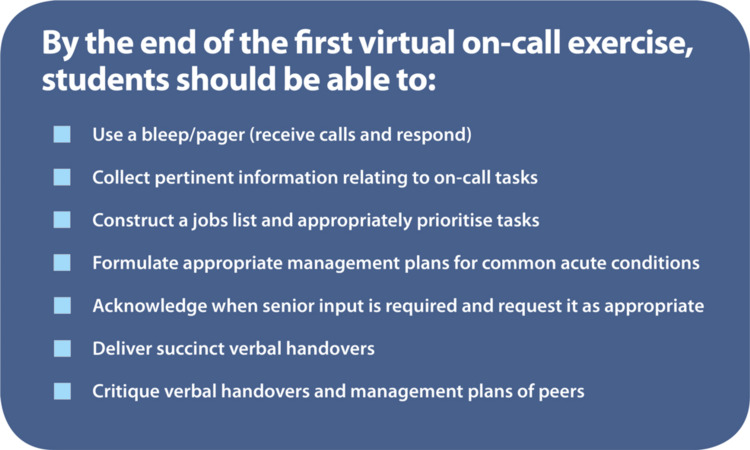
Virtual on-call intended learning outcomes

The intended learning outcomes are shown in Figure 1. Table 1 displays an overview of the program structure alongside the relevant educational theory.

**Table 1:** Details of teaching session stages with supporting educational theory QR code: quick response code

Session Stage	Stage Details and Supporting Educational Theory
Introduction	Students were familiarised with the Doctors’ Mess (i.e., Doctors’ Staff Room) and introduced to fellow participants as well as the facilitators. Housekeeping was completed and ground rules were developed in collaboration with the group. The pre-session questionnaire was completed. The intended learning outcomes were explained and students were asked if they had any specific learning needs or concerns. Students were shown how to use the bleep (also termed pager) and were briefly explained the hospital layout. Students were given the mobile number of their senior contact from whom they could ask for help. The introduction of all participants facilitates the creation of a learning community where all can contribute, ensuring all participation is encouraged and acknowledged, leading to more effective learning [6]. A clear introduction, including housekeeping, satisfies the basic levels of Maslow’s hierarchy of needs, attending to the basic psychological needs of students to create an environment that is conducive to learning [7]. The simulation was paper-based to reduce set-up/running costs, exclude the possibility of technological malfunction, remove the need for specific technological training, and improve reproducibility (including in limited resource settings) when compared to high-fidelity simulation such as that within a simulation suite. Real bleeps and ward locations were used to increase psychological fidelity and to better emulate the on-call environment. Collaborative discussion around ground rules (including confidentiality within the session and the ability to request a time-out mid-simulation) aids the creation of a supportive learning environment and helps to achieve psychological safety [8].
Simulated exercise	Students completed a series of five on-call tasks at different locations throughout the hospital. Faculty would bleep the students, playing the role of ward nurses or doctors, to handover tasks based on different wards. An example task may be "Doctor, I'm calling from ward 31. Bed eight has had a fall, I need you to come to review the patient." Students were required to obtain pertinent information about the task over the telephone before attending the relevant ward, for example, to ask what the observations were/ whether the fall was witnessed/if the patient was on anticoagulation. Upon arriving on the ward, they would find a folder containing simulated patient notes, including the admission clerking, drug chart, blood results, and the results of relevant investigations. The students would review the notes and write down how they would assess and manage the patient (see Figure 8 in Appendices) before continuing on to another ward to complete their next task. They completed the same tasks in different orders (see Figure 9 in Appendices). The details of the first were delivered via face-to-face handover in the Mess from the ‘day doctor’ with subsequent tasks communicated via the bleep. Participants could receive additional distractor bleeps. Example tasks may include: pain management, patient leaving against medical advice, death verification, hyperkalemia management, and management of a septic patient. Afterward, students returned to the Mess, where they each provided a verbal handover to the incoming ‘night doctor’. Such active, experiential learning aims to improve the effectiveness of the teaching through greater understanding, retention, and achievement of higher-order learning outcomes [9,10].
Debrief and feedback	Students participated in a facilitator-led reflective debrief of the exercise. Students additionally received peer and facilitator-led individualized feedback relating to their handover/performance. Students were asked to complete post-session questionnaires and questions were welcomed by faculty. A QR code linked to further educational resources relating to the on-call tasks was shared. Peer discussion within a varied group allows participants the opportunity to broaden the open areas of their Johari window [11]. As dictated by Kolb’s reflective cycle, an effective debrief can encourage consolidation and transformation of learning, particularly when learning points are applied to the clinical environment [12,13].

Student recruitment

All fourth and fifth year undergraduate medical students on clinical placement within Maidstone and Tunbridge Wells National Health Service (NHS) Trust were contacted via email inviting them to sign up for the voluntary sessions. The students, in their penultimate or final year of study, attended King’s College London and St George’s University of London.

Facilitator recruitment

Resident doctors of all grades were contacted via email inviting them to voluntarily sign up as a session facilitator or a site leader. Posters with quick response (QR) code sign-up links were also displayed in high-traffic areas such as the Doctors’ Mess. Facilitators were inducted by the site leaders and were supervised during their first session.

A live online document was used for facilitator and student session sign-up. A total of 30 resident doctors volunteered to teach during the sessions, with 93% (n=28) facilitating multiple sessions. Five doctors held site leadership roles. Around 39 students attended at least one teaching session.

Session logistics

VOC sessions took place on weekday evenings, typically lasting one to two hours. Sessions were delivered cross-site at Maidstone and Tunbridge Wells Hospitals. Prior to the session commencing, faculty placed patient notes (clearly marked for teaching use only) in the reception areas of wards and informed the ward staff of the simulation exercise. Students and faculty met in the Doctors’ Mess at the start and end of the sessions. Sessions could accommodate up to four students and required at least two resident doctor facilitators. Site leaders had oversight of the sessions and were responsible for encouraging sign-ups and supporting the facilitators.

Data collection and analysis

The primary outcome of the study assessed the change in confidence regarding various aspects of an on-call shift - prioritizing tasks, collecting information relating to the task, constructing a jobs list, managing acutely unwell patients, prescribing, accessing and using guidelines, seeking senior help and handing over - with results of five-point Likert scaled self-assessed questions compared between the study groups before and after the exercise. A secondary outcome was the difference in ratings following multiple sessions. The null hypotheses predicted no improvement in self-assessed confidence ratings following the session or further improvement following the attendance of multiple sessions.

Additional data were collected prior to the session on how the students felt about their first on-call and what their expectations of the teaching sessions were. Students were again asked how they felt about their first on-call shift following the exercise. The post-session questionnaire also captured qualitative data relating to the student learning points and involved a teaching evaluation.

Data were collected through anonymized online questionnaires (see Figures 10-14 in Appendices), accessed via a QR code, over a seven-month period. Forty-four pre-session responses were collected from 35 students: 27 attended one session, six attended two, and two attended three. Thirty-eight post-session responses were collected from 31 students: 24 students attended a single session, five attended two, and two attended three. Thirty-seven responses were paired using unique student codes and were obtained from 30 individual students: 23 individuals with paired responses attended a single session, five students attended two, and two attended three. Unpaired responses were excluded from paired data analysis.

The statistical significance of confidence differences between paired pre-session and post-session data was assessed with a paired t-test using GraphPad 2024 software (Dotmatics, Boston, USA). Statistical significance was considered at p<0.05. Qualitative data were thematically analyzed by two independent researchers.

The work was conducted in compliance with the ethical principles of the Declaration of Helsinki [14]. There was no potential harm to participants; the anonymity of participants is guaranteed. Anonymous data have been collected and stored in accordance with institutional data protection guidelines, and informed consent from participants was obtained for participation and publication. The project was approved locally by Maidstone and Tunbridge Wells NHS Trust (reference number QIP/02066). Data collected formed part of the quality assurance and service evaluation processes for medical education at the Trust.

## Results

Attitudes toward on-call working

**Figure 2:**
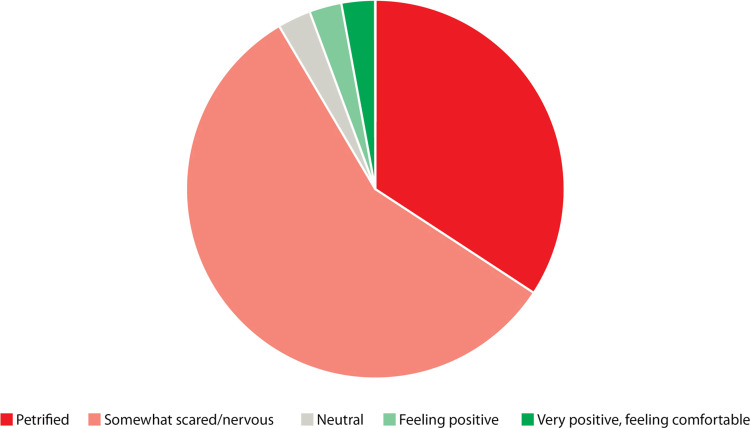
Participant self-rated attitudes toward their first on-call shift prior to their first VOC session (n=35) VOC: virtual on-call

The pie chart in Figure 2 displays the attitudes participants had toward their first on-call shift prior to their first VOC session. The chart displays an overwhelming majority (91%, n=32) expressing negative feelings of fear and nervousness.

**Figure 3:**
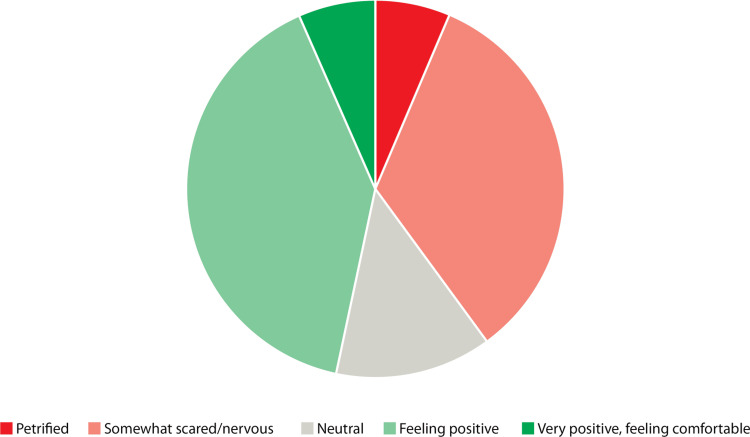
Participant self-rated attitudes towards their first on-call shift following to their first VOC session (n=30) VOC: virtual on-call

Figure 3 displays the attitudes participants had toward their first on-call shift following their first VOC session. Here, 40% (n=12) of participants still had negative feelings towards their first shift, while 47% (n=14) had positive attitudes.

Following the first VOC session, there was a statistically significant improvement in attitudes toward the first on-call shift, with a mean rating of somewhat scared/nervous for the first group and an average neutral rating for the second group (66% improvement in mean Likert rating, p<0.0001).

Prior to their second session, 86% (n=6) had negative feelings about their first on-call shift, a higher percentage than the post-first session survey. After the second session, half of the group (n=3) felt positive about their first shift, with the other half still feeling negative (n=3). Prior to the third session, 100% of participants expressed positive feelings (n=2).

Confidence ratings

**Figure 4:**
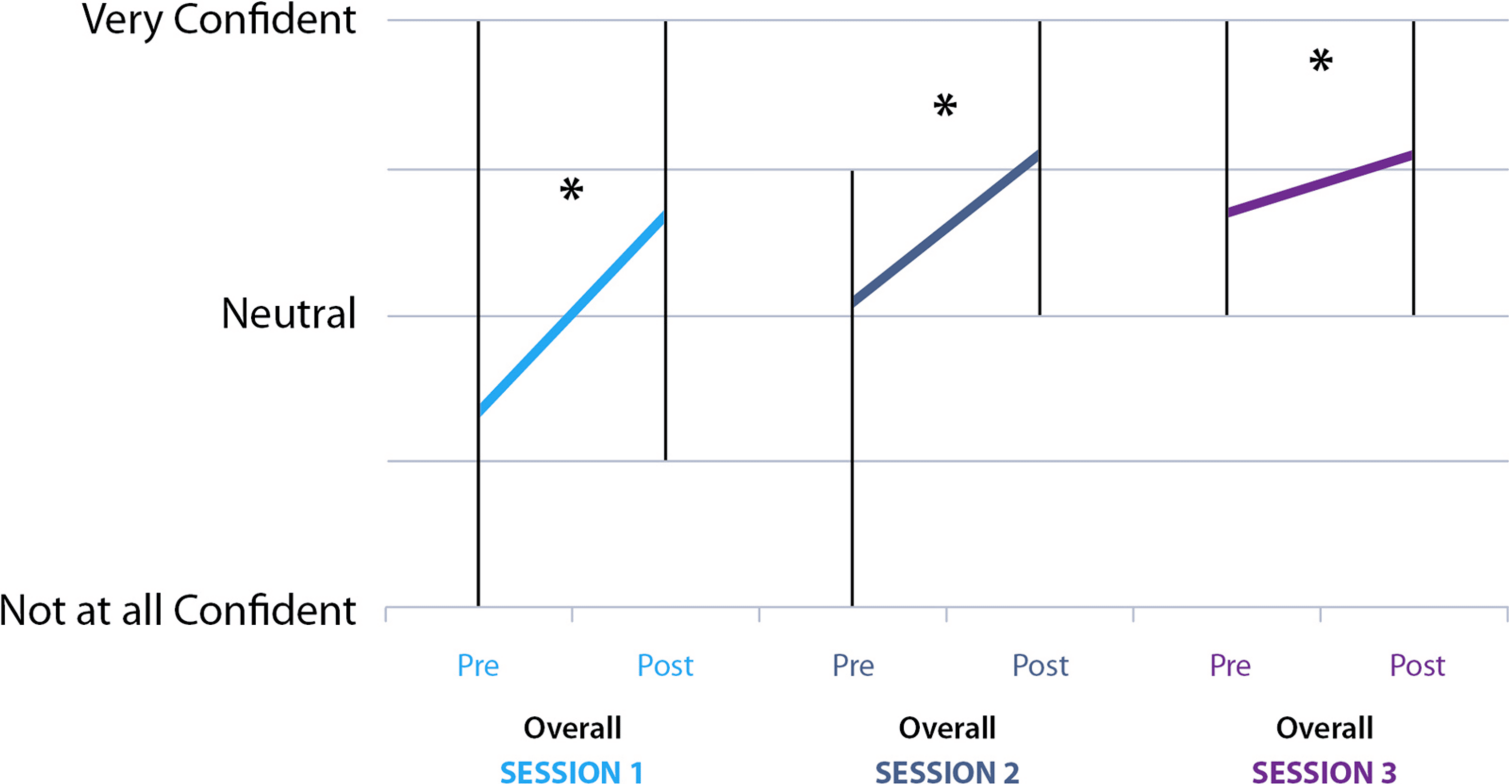
Pre- and post-session mean overall self-rated confidence (all on-call task ratings combined), divided by session (session 1 in light blue, session 2 in dark blue, session 3 in purple). Vertical bars denote the range. Asterisks denote statistically significant percentage increase in mean self-rated confidence following the session. First session (n=29 pairs): 54% increase, p<0.0001; Second session (n=6 pairs): 36% increase, p<0.0001; Third session (n=2 pairs): 12% increase, p=0.01

The graph in Figure 4 displays the overall self-rated mean confidence pre- and post-session for all components of the first on-call shift combined. The data is separated by the number of sessions attended.

**Figure 5:**
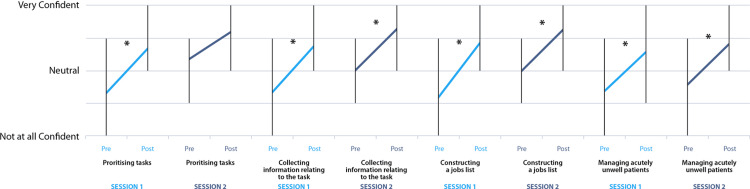
Pre- and post-session mean self-rated confidence with multiple on-call shift tasks (prioritizing tasks, collecting information, constructing a jobs list, and managing acutely unwell patients), divided by session (session 1 in light blue, session 2 in dark blue). Vertical bars denote the range. Asterisks denote statistically significant percentage increase in mean self-rated confidence following the session. First session (n=29 pairs): ‘Prioritizing tasks’ (62% increase, p<0.0001), ‘Collecting information’ (62% increase, p<0.0001), ‘Constructing a jobs list’ (88% increase, p<0.0001), ‘Managing acutely unwell patients’ (52% increase, p<0.0001); Second session (n=6 pairs): ‘Prioritizing tasks’ (21% increase, p=0.1), ‘Collecting information’ (39% increase, p=0.03), ‘Constructing a jobs list’ (39% increase, p=0.04), ‘Managing acutely unwell patients’ (49% increase, p=0.01)

**Figure 6:**
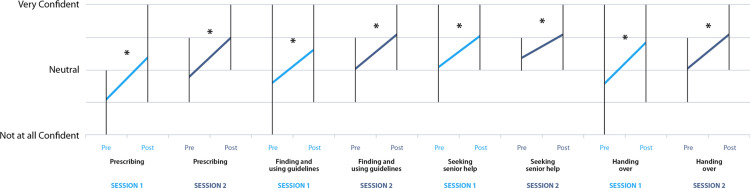
Pre- and post-session mean self-rated confidence with multiple on-call shift tasks, (‘Prescribing’, ‘Finding and using guidelines’, ‘Seeking senior help’ and ‘Handing over’), divided by session (session one in light blue, session two in dark blue). Vertical bars denote the range. Asterisks denote statistically significant percentage increase in self-rated confidence following the session: First session (n=29 pairs): ‘Prescribing’ (58% increase, p<0.0001), ‘Finding and using guidelines’ (37% increase, p<0.0001), ‘Seeking senior help’ (36% increase, p<0.0001), ‘Handing over’ (49% increase, p<0.0001). Second session (n=6 pairs): ‘Prescribing’ (47% increase, p=0.0009), ‘Finding and using guidelines’ (39% increase, p=0.0009), ‘Seeking senior help’ (22% increase, p=0.04), ‘Handing over’ (39% increase, p=0.04)

Figures 5-6 display this data in more detail, divided into the component on-call tasks assessed. Statistically significant percentage increases across all domains were seen between the pre- and post-session questionnaire for the first session (p<0.0001 for all domains). The largest effect in the first session was noted in confidence ‘Constructing a jobs list’, with an 88% increase in mean confidence (p<0.0001). This was followed by ‘Collecting information’ (62%, p<0.0001) and ‘Prioritizing tasks’ (62%, p<0.0001). The lowest confidence increase in session one, 36% (p<0.0001), was noted for ‘Seeking senior support’. For session two, statistically significant increases in mean confidence rating were seen for all domains except prioritizing tasks (21% increase, p=0.1). The largest effect for the second session was seen in ‘Managing acutely unwell patients’ (49% increase, p=0.01), with the smallest significant confidence increase being in ‘Seeking senior help’ (22% increase, p=0.04).

When comparing the paired pre-session self-rated confidence from the first and second sessions, there was a statistically significant increase overall for the combined on-call components (24% increase, p<0.0001).

Breaking this down by on-call duties, significant increases were seen across five domains: ‘Prioritizing tasks’ (50% increase, p=0.0004), ‘Collecting information’ (28%, p=0.02), ‘Constructing a jobs list’ (46% increase, p<0.005), ‘Finding and using guidelines’ (13%, p=0.045), and ‘Seeking senior help’ (14%, p=0.0038). For other domains, the increase in confidence was not statistically significant.

Teaching evaluation

Overwhelmingly positive feedback was received in the teaching evaluation survey after the sessions. Ninety-five percent of respondents (n=36) felt that the VOC session helped them to develop confidence in their own clinical decision-making skills (two responses stated that the session may have developed confidence).

Ninety-seven percent of respondents (n=37) felt the VOC sessions were useful in allowing them to simulate/assume the role of a foundation doctor (mean five-point Likert score of 4.66, with one being not at all useful and five being very useful; n=38) and 89% found their individual feedback to be useful (mean five-point Likert score of 4.42; n=38).

Ninety-five percent felt the sessions helped them with time management and working under pressure (mean five-point Likert score of 4.56; n=38). Despite the pressures of the session, students felt VOC provided a very constructive learning environment (mean five-point Likert score of 4.76; n=38), with all participants rating the learning environment as positive (n=36) or neutral (n=2). 

Thematic analysis of learning points revealed the most commonly mentioned was an improvement in prioritization and time management skills (referred to in 17 responses). Understanding when to escalate to seniors was the second most common learning point (nine responses) followed by the management of acutely unwell patients (eight responses). Other recurring themes included learning how to manage stress/pressure, communication skills, including how to hand over and escalate to seniors, and logistical skills such as using a bleep and navigating a hospital. Students felt they gained clinical knowledge regarding the management of acutely unwell patients and prescribing. Participants also provided positive qualitative feedback on the post-session online educational resources.

**Figure 7:**
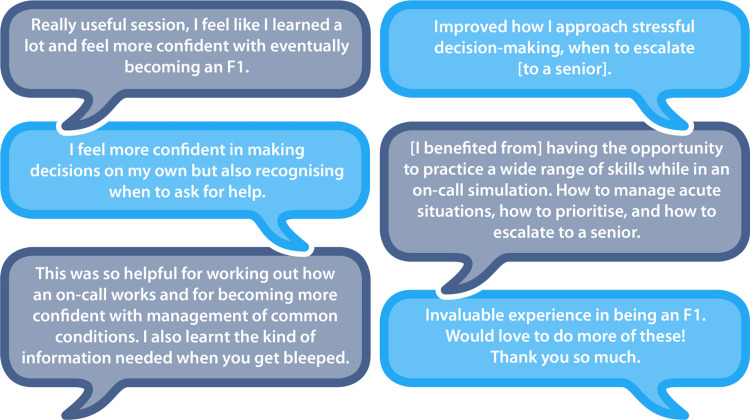
Examples of student white space responses to the question 'What did you gain from this session?'

**Figure 8:**
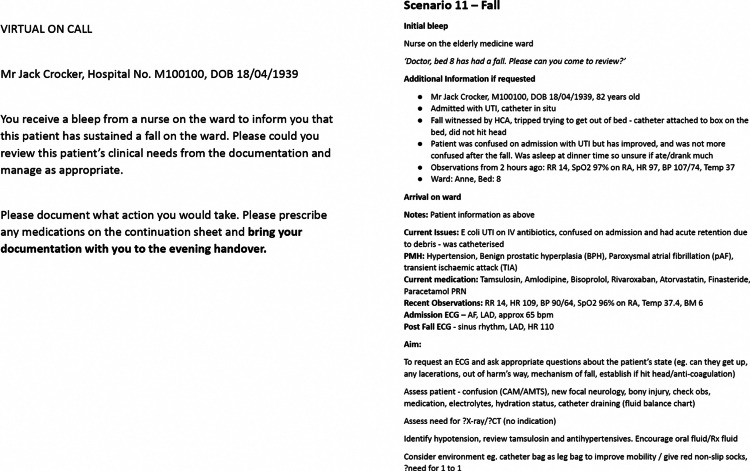
Example scenario (post-fall review)

**Figure 10:**
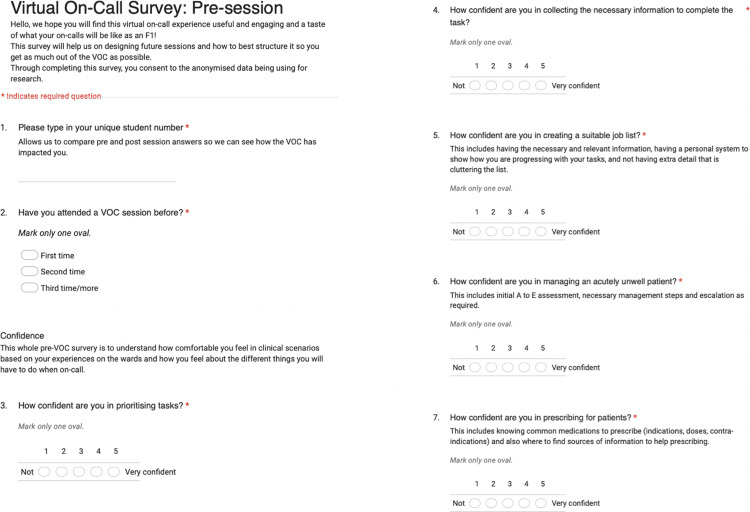
Pre-session survey 1/2 VOC: virtual on-call

**Figure 11:**
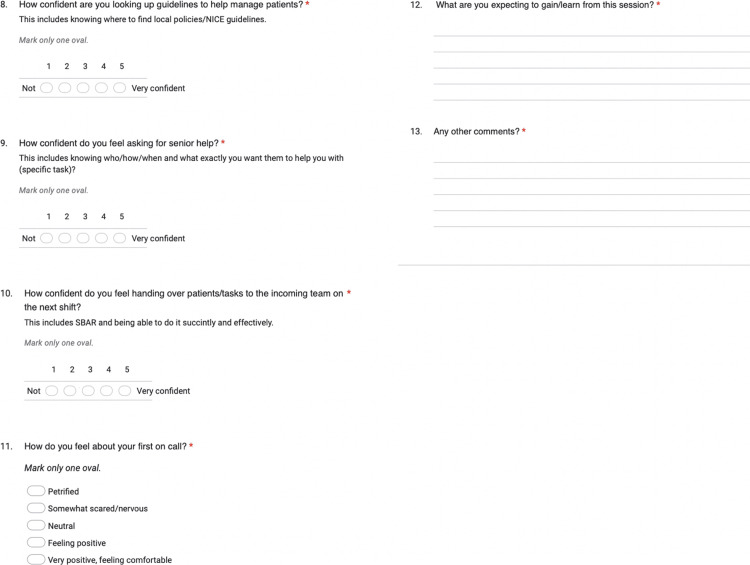
Pre-session survey 2/2 NICE: National Institute for Health and Care Excellence; SBAR: situation, background, assessment, recommendation

**Figure 12:**
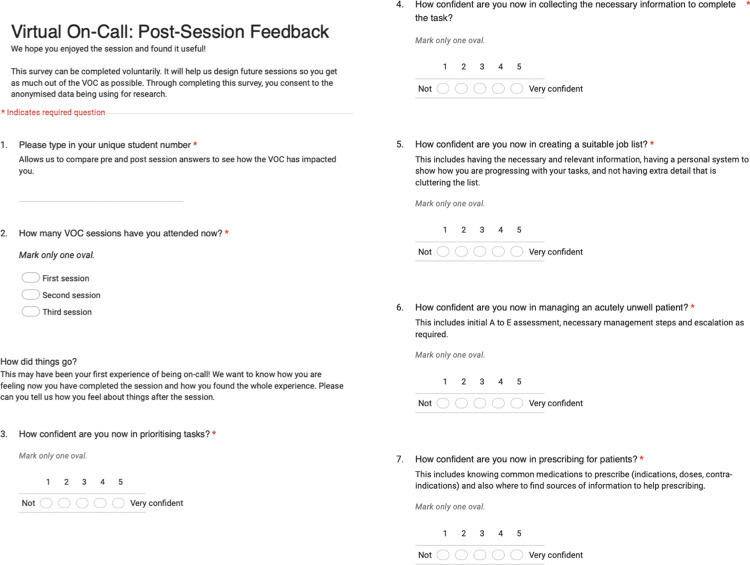
Post-session survey 1/3 VOC: virtual on-call

**Figure 13:**
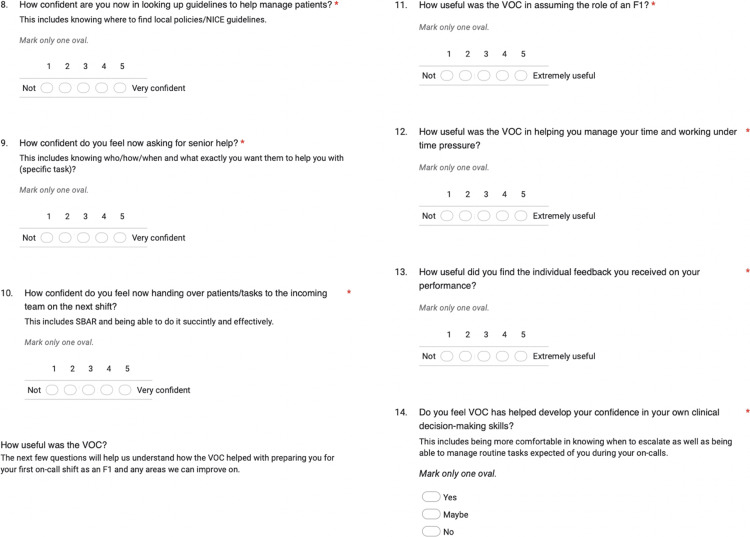
Post-session survey 2/3 VOC: virtual on-call; SBAR: situation, background, assessment, recommendation

**Figure 14:**
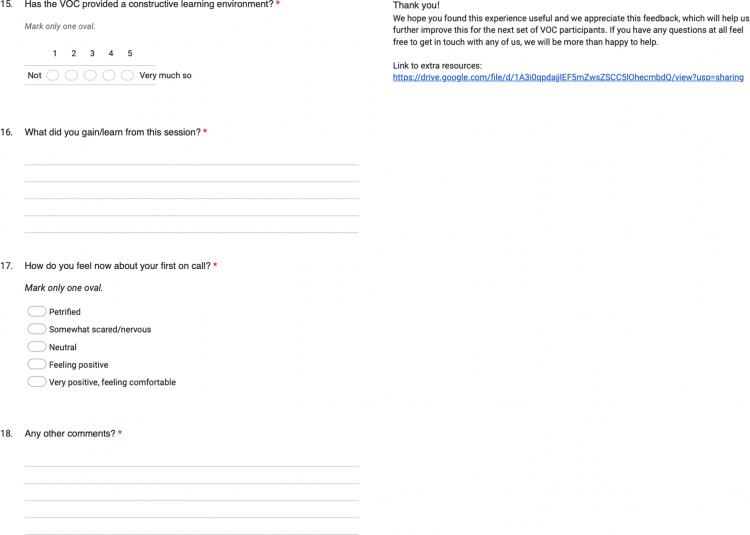
Post-session survey 3/3 VOC: virtual on-call

Figure 7 displays a selection of white-space student responses.

## Discussion

The General Medical Council (GMC) ‘Outcomes for Graduates’ stipulates that newly qualified doctors must be able to "demonstrate the clinical responsibilities and role of the doctor" [15]. With on-call shift work being commonplace in foundation trainee rosters, this implies that new graduates should be comfortable performing such on-call roles. The GMC guidelines outline how the medical schools, local education providers (such as associated Hospital Trusts), and the undergraduates themselves are responsible for ensuring individuals are "fit to practice safely as doctors when they graduate" [15].

Despite this GMC requirement, our study adds to mounting evidence highlighting a lack of confidence and preparedness among medical students regarding on-call shifts, suggesting an unmet educational need.

Although all student participants had completed several months of clinical placements as part of their undergraduate studies, the baseline needs assessment revealed a clear lack of confidence relating to on-call tasks. It is possible that reduced clinical exposure secondary to the COVID-19 pandemic may have played a role in this [16]. Nonetheless, it is essential that local curricula followed by medical schools and local education providers are designed to prepare students sufficiently for on-call working.

This study reveals the negative emotions such as fear and nervousness that students feel regarding these shifts. It is widely known that fear, stress, and anxiety can negatively impact the mental health of individuals, increasing the risk of burnout and absenteeism in the long term [17]. Such emotions are also known to impact workplace performance and can lead to higher rates of medical errors [18,19]. Thus, it is essential that students are well supported in their transition to the foundation program and prepared for the more anxiety-inducing shifts such as on-call duties.

It was encouraging to see increases in positive feelings towards the first on-call shift following the VOC session, suggesting this is an effective educational tool in reducing such feelings of anxiety. It is interesting, however, to note that the percentage of participants with positive emotions dropped in the cohort prior to the second session. There may be some selection bias as those who remained under-confident may have been more likely to sign up for additional sessions to further develop their skills and confidence. There may also be some natural variation in confidence over time or alterations in student perceptions as they gain a more realistic view of on-call work. The data could suggest the session provides only reassurance, with further sessions being required to reinforce their positive emotions and confidence. Further research into the effect of multiple sessions would be of benefit.

Our data reveal significant overall increases in confidence in relation to the on-call over the first and second sessions, with data for third-session attendees being limited by participant number. It is rewarding to see the mean confidence increase for repeat attendees, displaying how the learning potential and benefits of the session continued to grow (Figure 4). It was interesting to see the domains with the largest increases in confidence differ between first- and second-session attendees, which may be in part due to variations in tasks between sessions.

The simulation was designed to test skills from across all three GMC outcomes (professional values and behaviors, professional skills, and professional knowledge) [14]. As stipulated in the overarching outcome (GMC), new doctors are required to "(apply) their knowledge and skills in a competent, ethical, and professional manner and taking responsibility for their own actions in complex and uncertain situations" [15].

The use of authentic psychological stressors, such as undifferentiated bleeps, time pressure, and tasks set in different physical locations, allowed us to effectively emulate the complex and uncertain situations that are encountered when on-call as a new doctor. The qualitative data reinforced this showing how despite the on-call being low-resource comprising paper-based activities, it had high psychological fidelity with students commenting on feelings of being under pressure and stressed.

Despite these psychological stressors, the teaching evaluation highlighted that the VOC sessions provided a constructive learning environment, with students reflecting positively on working within a more stressful environment, within the scaffolded teaching session. Students commented on how the sessions allowed them to “(learn) so much in relation to how an F1 feels on-call."

The flexibility of these sessions allowed us to test student responses and support those who required more guidance or challenge those who were managing well. Students who were excelling within the simulation or those who were repeat attendees could be tested by introducing the need for more advanced communication skills over the telephone, i.e., with angry referrers and increasing the number of distractor bleeps.

It is encouraging to see how previous studies of a similar nature, albeit involving far fewer participants, also showed the efficacy of such paper-based simulations in improving preparedness for practice among medical students, reinforcing the value of such teaching program designs [20]. Interestingly, the literature reinforces the concept of low-fidelity simulation not being inferior to high-fidelity simulation, with some studies in fact finding that higher-fidelity simulation can lead to a sense of over-confidence among medical students [21].

In addition to aiding student participants, the VOC benefitted resident doctors, as evidenced by the high facilitator sign-up rate, with 93% teaching on multiple sessions. VOC provided career development opportunities by enabling resident doctors to gain teaching and leadership experience with valuable peer and student feedback. 

Limitations

Facilitator inductions, leader supervision, and uniform learning materials provided a level of quality assurance across the sessions; however, some variation in teaching quality between facilitators is unavoidable. The generalizability of this study is limited by the relatively small number of participants, which may be partly attributed to the voluntary nature of session attendance and session timings in the evening. The low response rate may additionally result in selection bias. This is particularly marked for the sample sizes of second- and third-time attendees. This being said, the delivery of sessions across two sites and the recruitment of students from two different medical schools do provide support for the reproducibility of these results.

## Conclusions

On-call work forms a key part of the foundation doctor's job role. This study adds valuable evidence to the existing literature highlighting the lack of confidence and fear of on-call working among medical students. As such feelings can catalyze clinician burnout and impair performance, it is essential that medical students are well-prepared for such shifts. The VOC provides a simple, low-resource, paper-based model that has been shown to significantly increase medical student confidence regarding on-call shifts and has the added benefit of providing resident doctors with teaching and leadership opportunities.

Following on from the success of this teaching initiative, the model has become established within the Trust and has been rolled out for subsequent year groups. Given its sustainability, low running costs, and importantly, efficacy, this teaching initiative has the potential to be replicated at Trusts nationwide, with the scope to adapt the sessions to cater to different specialties and training levels. Future work to evaluate the long-term impact on participants' confidence and clinical performance upon commencing foundation training would be of benefit.

## Figures and Tables

**Figure 9:**
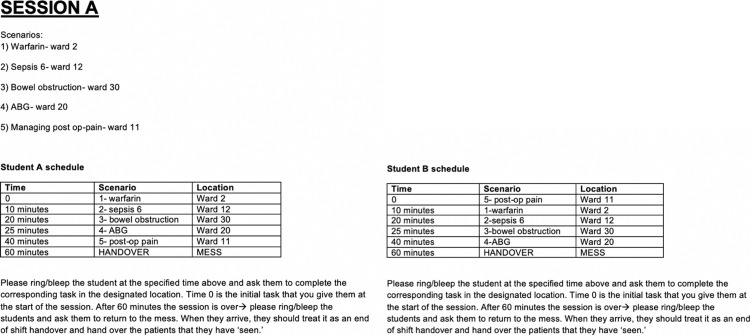
Example student schedule
